# Endogenous and exogenous sex steroid hormones in asthma and allergy in females: protocol for a systematic review and meta-analysis

**DOI:** 10.1038/npjpcrm.2015.78

**Published:** 2016-01-28

**Authors:** Bright I Nwaru, Ulugbek Nurmatov, Aziz Sheikh

**Affiliations:** 1 Asthma UK Centre for Applied Research, Centre for Medical Informatics, Usher Institute of Population Health Sciences and Informatics, The University of Edinburgh, Edinburgh, UK; 2 School of Health Sciences, University of Tampere, Tampere, Finland

## Background

Before puberty, asthma and allergy are more common in males than in females, but these conditions become more common in females than in males during adulthood until around the time of menopause.^1–7^ The disease severity, healthcare utilisation and impact on health-related quality of life (HRQoL) are also considerably higher in post-pubertal females than in males.^1–7^ Although the specific mechanisms for these differences are unclear, it has been suggested that female sex steroid hormones may have a role.^1,2^ Higher disease risk in females appears to follow key hormonal transitional time points in a female’s reproductive life cycle, such as puberty, menarche, menstruation and menopause.^1,2^ These hormonal transitional points may increase the risk of asthma and poor asthma outcomes in females.^8–10^ In contrast, external suppression of endogenous sex hormone production with hormonal contraceptives may improve asthma outcomes,^9–14^ but the evidence is inconsistent,^13,14^ with evidence suggesting that hormone replacement therapy (HRT) may increase the risk of poor outcomes.^15–22^

A clearer understanding of the role of sex steroid hormones in the development of asthma and allergy in females and their impact on asthma exacerbation, use of healthcare, and HRQoL will help to provide new insights into disease mechanisms and essential preliminary data to inform the development of primary prevention interventions and hormonal-based management strategies. We plan to undertake a systematic synthesis of the evidence to provide a comprehensive, unbiased estimate of the actual effects of sex steroid hormones on the development and clinical expression of asthma and allergy in females.

The only relevant previous systematic review on this topic was confined to investigating the role of menopausal transition; this review focussed on asthma incidence as the sole outcome of interest.^23^ Consequently, that study does not allow a thorough appraisal of this evidence base, given that key endogenous and exogenous hormonal factors, as well as other essential asthma and allergy outcomes, were not considered. There is therefore the need to undertake a more comprehensive synthesis of the effect of the various endogenous and exogenous hormonal factors on the full spectrum of asthma outcomes in females.

## Aims and objectives

We seek to identify, critically appraise, and undertake meta-analyses of the evidence on the role of endogenous and exogenous sex steroid hormones in the development and clinical expression of asthma and allergy in females, in addition to synthesising the evidence on the effectiveness of hormonal contraceptives and HRT for the secondary prevention of asthma. Specific primary objectives are to synthesise and interpret the evidence on the role of:

1. Puberty, menarche, menstruation and menstrual-related changes, and menopause in the development of asthma and allergy and their impact on asthma exacerbation, hospitalisation, airway function and HRQoL in females.
2. Hormonal contraceptives and HRT and their subtypes in the development of asthma and allergy and their impact on asthma exacerbation, hospitalisation, airway function and HRQoL in females.

Our secondary objective is to synthesise and interpret the evidence on the potential effectiveness of hormonal contraceptives and HRT for the secondary prevention of asthma in females.

## Methods

### Types of studies

We will include the following studies: all experimental studies, including randomised controlled trials, quasi-randomised controlled trials, controlled-clinical trials, controlled before and after studies, and interrupted time series design; cohort studies; case–control studies; and cross-sectional studies. Reviews, case studies and case series, and animal studies will, however, be excluded. For evidence on the effectiveness of hormonal contraceptives and HRT for the secondary prevention of asthma, we will consider only randomised controlled trials.

### Participants

The participants will include all females from puberty to adulthood. Recognising that age at puberty is defined differently across cultural contexts, we will have no fixed lower age limit for puberty for this review but will adopt definitions as given in each paper. The age limit for adulthood will be 75 years.

### Types of exposure

We will include studies that have investigated the role of puberty, menarche, menstruation, hormonal contraceptives (combined oral contraceptives and progesterone-only preparations), menopause (pre- and post-menopause, natural and surgical menopause) and HRT (oestrogen-only, progesterone-only, and combined oestrogen–progesterone preparations).

### Outcome measures

#### Primary outcomes

Our primary outcomes will include self-reported or objectively defined incidence and prevalence of asthma, asthma exacerbation, hospitalisation and use of asthma medication.

#### Secondary outcomes

Secondary outcomes will include the following: self-reported or objectively defined incidence and prevalence of wheezing, atopic dermatitis/eczema, allergic rhinitis, urticaria, angio-oedema, food allergy, anaphylaxis and atopic sensitisation; indicators of airway function (forced expiratory volume in 1 s, forced expiratory flow, forced vital capacity, peak expiratory flow); and measures of HRQoL.

### Search strategies to identify studies

We will employ a highly sensitive search strategy to retrieve articles meeting the review criteria. Supplementary Appendix 1 contains the search strategies developed for MEDLINE database interfaces, which we will adapt to search other databases. Databases to search in retrieving relevant papers will include the following: MEDLINE, EMBASE, Cochrane Library, ISI Web of Science, CINAHL, Scopus, Google Scholar, AMED, PsychINFO, CAB International and WHO Global Health Library. The databases will be searched for studies published between 1990 and 2015. Additional references will be located through searching the references cited in identified papers, as well as searching databases of the proceedings of international conferences, such as ISI Conference Proceedings Citation Index and ZETOC (British Library). We will locate unpublished and ongoing studies by searching trial registries, including Current Controlled Trials (http://www.controlled-trials.com), ClinicalTrials.gov (http://www.clinicaltrials.gov) and Australian and New Zealand Clinical Trials Registry (http://www.anzctr.org.au), and by contacting a panel of international experts in the field. There will be no language restrictions, and where possible we will translate literature in languages other than English and report any literature we are unable to translate.

### Screening of studies

Titles and abstracts of the retrieved articles will be checked independently by two reviewers according to the review criteria; any discrepancies will be resolved by consensus, and disagreements will be arbitrated by a third reviewer. Full-text copies of potentially relevant studies will be obtained and their eligibility for inclusion independently assessed by two reviewers; discrepancies will be resolved by consensus, and disagreements will be arbitrated by a third reviewer.

### Data extraction and reporting

Two reviewers will independently extract relevant information and study data onto a customised data extraction sheet, which will initially be piloted and revised where necessary using a sample of a few of the eligible studies. Any discrepancies in data extraction will be resolved by discussion or arbitration by a third reviewer if agreement cannot be reached. Descriptive tables will be used to summarise the literature and characteristics of studies contributing to the overall evidence. The role of each of the endogenous (puberty, menarche, menstruation and menopause) and exogenous (hormonal contraceptives and HRT) sex steroid hormones in the risk of the study outcomes will be reported separately. We will use the PRISMA checklist to guide the reporting of the review.^24^

### Quality assessment and risk of bias

The quality of eligible studies and the potential for risk of bias will be independently undertaken by two reviewers; any discrepancies will be resolved by discussion or arbitration by a third reviewer. We will assess the methodological quality of experimental studies based on the recommendations of the Cochrane Handbook for Systematic Reviews of Interventions as well as the Cochrane Effectiveness and Practice Organisation of Care (EPOC) guidelines.^25^ The recommendations highlight seven domains of focus in appraising intervention studies: sequence generation, allocation concealment, blinding of participants and personnel, blinding of outcome assessment, incomplete outcome data, selective outcome reporting and other sources of bias.^25^ We will derive domain-specific and overall quality grading for each study as follows: A—low risk of bias; B—moderate risk of bias; C—high risk of bias. We will use the Effective Public Health Practice Project (EPHPP) (www.ephpp.ca) to quality-appraise observational studies and will also derive component-specific (i.e., suitability of the study design for the research question; risk of selection bias; exposure measurement; outcome assessment; and generalisability of findings) and overall grading for each study.

### Data syntheses

We will undertake a narrative synthesis of the data. In addition, for studies deemed to be reasonably clinically, methodologically and statistically homogeneous, we will perform meta-analyses using random-effects models. In comparison with fixed-effect meta-analysis, the random-effects model is a more conservative approach as its underlying assumption is closer to reality, particularly synthesising studies obtained only from the published literature, and it incorporates potential heterogeneity between studies when calculating the pooled estimates.^26^ We will quantify the heterogeneity between studies using the *I* ^2^ statistic. The meta-analyses will be performed separately for experimental and observational studies and within each study design will also be undertaken separately for groups of studies falling into each type of sex steroid hormonal exposure (puberty, menarche, menstruation, hormonal contraceptives, menopause and HRT). Where possible, we will perform subgroup analyses according to females’ background characteristics, such as age, body mass index, smoking and other potential characteristics. We will perform sensitivity analyses on the basis of risk of bias in the studies in order to evaluate the robustness of our findings. We will assess evidence of publication bias using funnel plots and statistically using Begg and Egger tests.^27,28^ The meta-analyses will be performed using Stata Statistical Software (Release 13; StataCorp LP., College Station, TX, USA).

### Protocol registration

A detailed protocol for the review will be registered with the International Prospective Register of Systematic Reviews (PROSPERO): http://www.crd.york.ac.uk/prospero/ prior to commencing the review.

## Discussion

The current review will present the most comprehensive and unbiased synthesis of the evidence relating endogenous and exogenous sex steroid hormones to the development and clinical expression of asthma and allergy in females. The only previous review on the topic considered menopausal transition as the only hormone exposure and asthma as the only outcome and therefore does not give a clear picture of the overall evidence base.^23^ Key strengths of the current review include the following: (1) synthesis of the role of both endogenous and exogenous sex steroid hormones; (2) presentation of the evidence for each key hormonal transitional point and exposure (puberty, menarche, menstruation, use of hormonal contraceptives, menopause and use of HRT) during the female’s life course and assessment of how each of these exposure definitions influences the risk of asthma in females; and (3) a comprehensive and highly sensitive search strategy and inclusion of known leading databases and a panel of expert contacts that will ensure that the main sources of retrieving literature for the underlying evidence are covered.

In ascertaining whether endogenous and exogenous sex steroid hormones have any role in the development and clinical expression of asthma and allergy in females, we aim to uncover the following: (1) putative role and critical time windows of the effect of each endogenous sex steroid hormone (puberty, menarche, menstruation and menopause); (2) effectiveness and safety of exogenous hormonal contraceptives and HRT and their subtypes as secondary prevention strategies; (3) subgroups of females who are more likely to benefit or could be at greater risk of asthma as a consequence of their sex steroid hormonal status or exposure; and (4) key research gaps in this evidence base, recommending steps to strengthen future work. Along these lines, the evidence from this study will in particular form the basis for initiating intervention trials to establish whether hormonal contraceptives and HRT can be effective in improving outcomes of asthma and allergic disorders in females.

### Conclusion

Synthesising the evidence that has so far emerged on the role of sex steroid hormones in the development and clinical expression of asthma and allergy in females will help to clarify the reason for the observed sex differentials in the onset and overall burden of asthma after puberty. Furthermore, a comprehensive synthesis of the evidence base will provide the opportunity to identify the potentials of exogenous sex steroid hormones for the primary and secondary prevention of asthma in females.

## Notes

AS is Joint Editor-in-Chief of *npj Primary Care Respiratory Medicine*, but was not involved in the editorial review of, nor the decision to publish, this article. The remaining authors declare no conflict of interest.
