# Supplementary material for: Endogenous and exogenous sex steroid hormones in asthma and allergy in females: protocol for a systematic review and meta-analysis
Source: NPJ Prim Care Respir Med. 2016 Jan 28;26:15078–. doi: 10.1038/npjpcrm.2015.78 (PMC4730990; doi:10.1038/npjpcrm.2015.78)
Supplement: Supplementary Information [file npjpcrm201578-s1.doc]

**Appendix 1: MEDLINE Search Strategy**

1. exp Puberty/ or puberty.mp.
2. exp Menarche/ or menarche.mp.
3. exp Menstruation/ or exp Menstruation Disturbances/ or menstruation.mp.
4. exp Menopause, Premature/ or exp Postmenopause/ or menopause.mp.
5. sex hormones.mp. or exp Gonadal Steroid Hormones/ or exp Estrogens/ or estrogens.mp. or exp Progesterone/ or progesterone.mp. or Testosterone Congeners/ or testosterone.mp. or exp Testosterone/ or Testosterone Propionate/
6. exp Contraceptive Agents/ or contraceptives.mp. or exp Contraceptives, Oral/ or oral contraceptives.mp. or exp Contraceptives, Oral, Combined/ or combined oral contraceptives.mp. or exp Medroxyprogesterone Acetate/ or exp Contraceptive Agents, Female/ or exp Contraceptives, Oral, Hormonal/ or exp Contraception/ or hormonal contraceptives.mp. or exp Ethinyl Estradiol/
7. exp Hormone Replacement Therapy/ or exp Estradiol/ or hormone replacement therapy.mp.
8. 1 or 2 or 3 or 4 or 5 or 6 or 7
9. exp Asthma/ or asthma.mp.
10. wheeze.mp.
11. exp Dermatitis, Atopic/ or atopic eczema.mp.
12. exp Hypersensitivity, Immediate/ or exp Hypersensitivity/ or atopy.mp. or allergy.mp. or atopic sensitisation.mp. or allergic sensitisation.mp.
13. exp Rhinitis, Allergic, Seasonal/ or exp Rhinitis, Allergic, Perennial/ or exp Allergens/ or allergic rhinitis.mp.
14. exp Conjunctivitis, Allergic/ or Rhinoconjunctivitis.mp.
15. exp Urticaria/ or urticarial.mp.
16. exp Angioedema/ or angioedema.mp.
17. exp Food Hypersensitivity/ or food allergy.mp.
18. exp Anaphylaxis/ or anaphylaxis.mp.
19. lung function.mp.
20. airway function.mp. or exp Bronchial Hyperreactivity/
21. exp Forced Expiratory Volume/ or forced expiratory volume in 1 second.mp.
22. forced expiratory flow.mp. or exp Vital Capacity/ or forced vital capacity.mp.
23. exp Peak Expiratory Flow Rate/ or peak expiratory flow.mp.
24. 9 or 10 or 11 or 12 or 13 or 14 or 15 or 16 or 17 or 18 or 19 or 20 or 21 or 22 or 23
25. 8 and 24
26. Limit 25 to female
